# Study in circular auxetic structures for efficiency enhancement in piezoelectric vibration energy harvesting

**DOI:** 10.1038/s41598-020-73425-1

**Published:** 2020-10-01

**Authors:** Pejman Eghbali, Davood Younesian, Armin Moayedizadeh, Mostafa Ranjbar

**Affiliations:** 1 School of Railway Engineering, Iran University of Science and Technology, 16846-13114 Tehran, Iran; 2 Department of Mechanical Engineering, Ankara Yildirim Beyazit University, Ankara, Turkey

**Keywords:** Energy science and technology, Energy harvesting, Mechanical engineering

## Abstract

Piezoelectric (PZT) components are one of the most popular elements in vibration sensing and also energy harvesting. They are very well established, cost effective and available in different geometries however there are still several challenges in their application particularly in vibration energy harvesting. They are normally narrow-band elements and work in high-frequency range. Their efficiency and power extraction density are also generally low compared with different electromagnetic techniques. Auxetic structures are proposed here to enhance efficiency of the piezoelectric circular patches in vibration energy harvesting. These kinds of patches namely PZT buzzers are inexpensive (less than 10 USD) elements and easily available. Two novel circular auxetic substrates are proposed to improve power extraction capacity of the conventional piezoelectric buzzers. Negative Poison’s ratio of the proposed meta-structure helps in efficiency enhancement. The concept is introduced, analyzed and verified through the finite element modeling and experimental testing. The idea is proved to work by comparing the harvested electrical power in the auxetic design against the conventional plain system. A parametric study is then carried out and effects of important electrical and geometrical parameters as well as the material property on the power extraction efficiency are assessed to arrive at optimum parameters. It is shown that by employing the auxetic design, a remarkable improvement in the harvested power is achievable. It is shown that for the two proposed auxetic designs, at the resonance frequency, we could reach to 10.2 and 13.3 magnification factor with respect to the plain energy harvester. Another important feature is that the resonant frequency in these new designs is very much lower than the conventional resonators. Results of this study can open a new path to application of inexpensive PZT buzzers in large-scale vibration energy harvesting.

## Introduction

Energy is a key factor in the modern smart world. Sensors are essential elements in smart maintenance and online monitoring systems. Powering these sensors and other electronic devices in remote and inaccessible places is still an engineering challenge. One attractive proposed solution is energy harvesting devices which could generate electrical power from different sources such as vibration^1–3^, wind^4,5^, sound^6,7^, ambient light^8,9^, temperature gradient^10,11^ and waves^12,13^. Energy harvesting from the ambient vibration in different environments is an active line of research. Conversion of the vibration energy to electricity is mainly achievable through three popular methods namely electromagnetic^14–16^, triboelectric^17–19^ and piezoelectric^20–23^. Piezoelectric elements have been widely utilized in vibration energy harvesting so far^24–26^, however, the low power and low efficiency of the PZT based energy harvesters is still a main challenge. Enhancement of these limitations is mainly attained through material science by changing the composition of piezoelectric elements^27,28^ or by structural solutions^29,30^ consist of metamaterials.

Emergence of metamaterials introduced an efficient way for piezoelectric power and efficiency enhancement. By expanding laterally under extension and contracting under compression, metamaterial cellular structures show negative Poisson’s ratio^31^. This abnormal behavior is achievable from the geometry of the metamaterial cellular structure. Different geometries can be proposed to have negative Poisson’s ratio called “Auxetic feature”^32–34^. The idea of metamaterial is inspired by nature, as it is seen in the structure of some woods, bones and hard tissue of some animals. In engineering applications, it has been proved that these structures have a promising performance in shock and impact absorption^35,36^, improving the bending stiffness of thin-walled structures^37^ and vibro-acoustics performance of sandwich panels^38,39^.

Exploring the auxetic feature in energy harvesting is a relatively new (since 2017) line of research^40–43^. PZT buzzers are available, universal and low-price elements which are widely used for different functions. Similar to other types of PZT patches, their performance in vibration energy harvesting is not great. With no need to change the type, size and material properties of the conventional buzzers, we are proposing two novel circular auxetic substrates to substantially improve their performance. Performance of these auxetic designs is compared to the conventional plain resonators with the same size and material. Finite element modeling and experiments prove the concept. Here, we will show that, the two auxetic harvesters can improve the output electrical power of piezoelectric buzzers with a magnification factor of $10.2$ and $13.3$ compared to the plain harvester. The idea presented here is a simple solution to use inexpensive PZT buzzers in large-scale vibration energy harvesting. This concept can remarkably reduce the cost of power and simultaneously enhance the level of available power in self-powered multi-sensor monitoring systems.

## Auxetic structure design strategy

**Figure 1:**
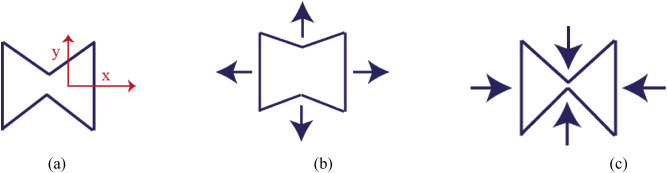
Two-dimensional honeycomb auxetic structure **(a)** free mode, **(b)** lateral (y direction) expansion under uniaxial tension in the x direction, **(c)** lateral (y direction) compression under uniaxial pressure in the x direction.

**Figure 2:**
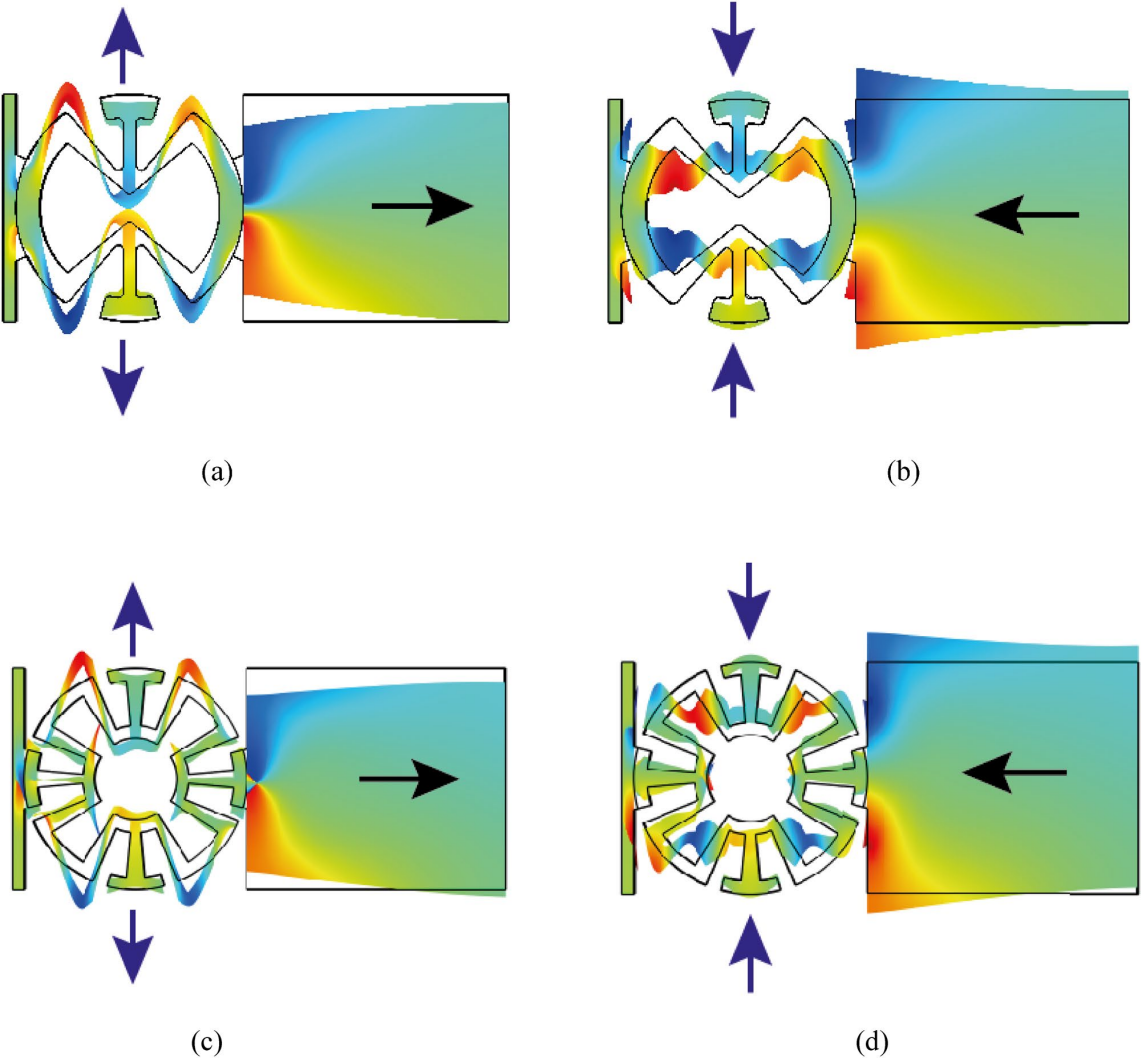
Proposed circular auxetic layouts, deformed shapes in colored presentation **(a)**, **(c)** lateral expansion of the auxetic region under uniaxial tension **(b)**, **(d)** lateral compression of the auxetic region under uniaxial pressure.

**Figure 3:**
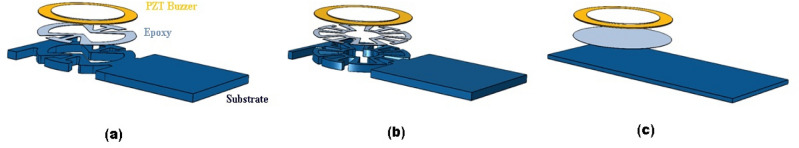
Cantilever beam energy harvester with auxetic structures **(a)** AEH-I, **(b)** AEH-II, **(c)** PEH.

Against the normal materials, auxetic structures get laterally expanded under uniaxial tension and laterally squeezed under axial compression. This is the key factor in a successful auxetic structure design. Among the first proposed auxetic structures one can refer to the two-dimensional honeycombs, from which a unit cell is shown in Fig. 1a. By applying a uniaxial tension in the longitudinal direction, it will be expanded laterally as shown in Fig. 1b, and it will be compressed laterally under uniaxial pressure in x direction (Fig. 1c). This means that the elements of the auxetic structure should be arranged in such a way to result in negative poison’s ratio. This idea is applied in this paper particularly for the circular layouts. Figure 2 shows the abnormal negative poison’s ratio behavior of the proposed circular auxetic designs. Figure 2a,c illustrate the lateral (y direction) expansion of the auxetic structures under uniaxial tension in the x direction. It is seen that by applying tension, inclined ligaments tend to move out of the circle. Figure 2b,d show the lateral compression under uniaxial pressure in the x direction. These two original auxetic geometries have been specially designed for the PZT buzzers which are available in circular shapes (Fig. 3).

## Results

### Circular auxetic structures

**Figure 4:**
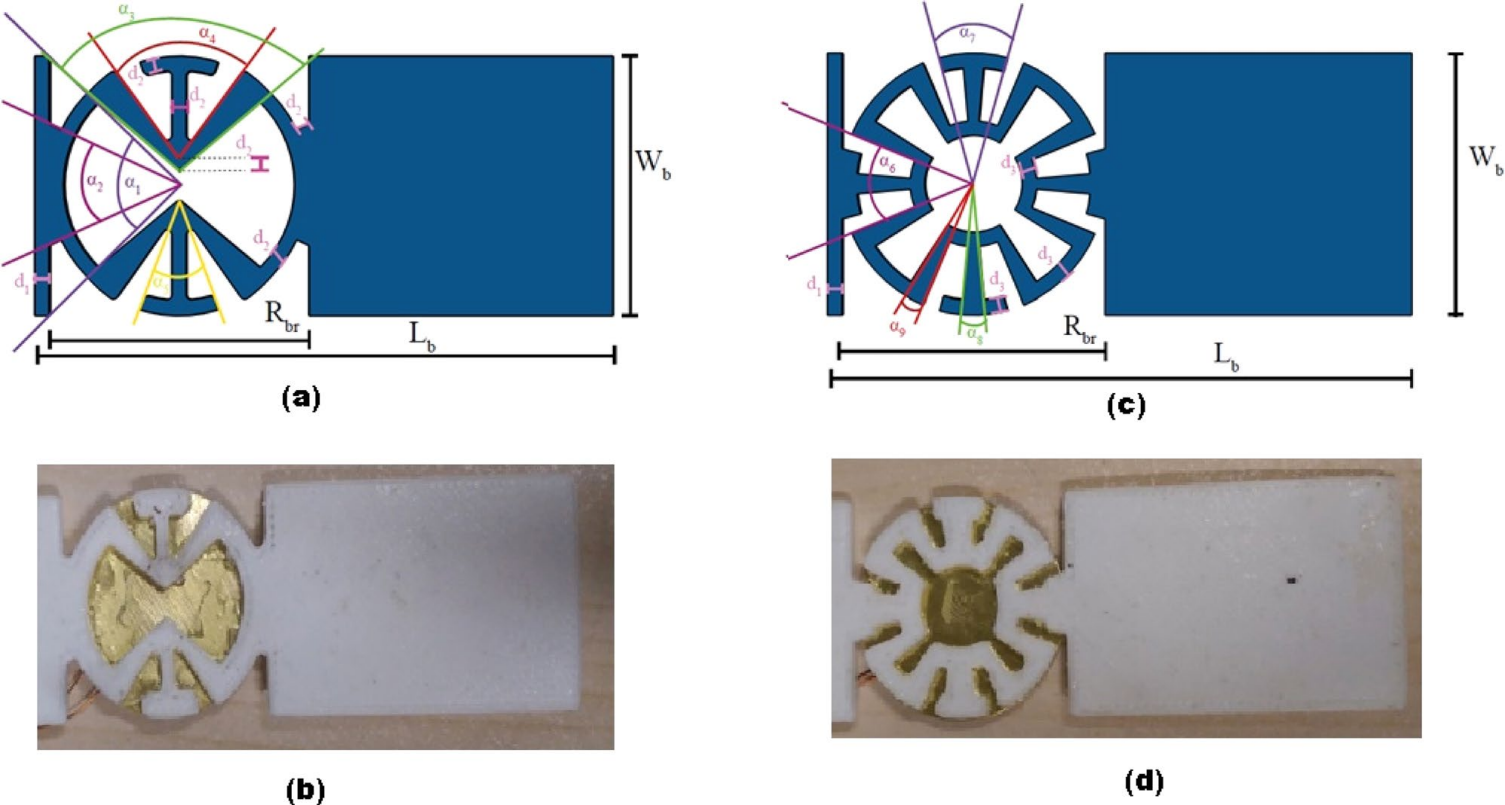
Geometry of the auxetic structure **(a)** AEH-I (schematic), **(b)** AEH-I (fabricated), **(c)** AEH-II (schematic), **(d)** AEH-II (fabricated).

We propose two novel circular auxetic structures (I and II) by which a magnificent enhancement is achievable in energy harvesting from circular piezoelectric elements (piezo buzzers). The auxetic structure will be at the very beginning of the cantilever beam, on which the PZT buzzer is attached (Fig. 3a,b). Performance of these auxetic energy harvesters (AEH) will be compared to a plain resonator called plain energy harvester (PEH) (Fig. 3c). Geometrical parameters of the lattice and fabricated auxetic beams are illustrated in Fig. 4a–d.

The reason of utilizing auxetic structures is behind two main features of them; (1) The piezoelectric element will be stretched in two perpendicular directions at the same time and vice versa. Taking the following equation into consideration, the importance of this feature will be emphasized^42^

1 $$power \propto {\left({\sigma }_{11}+{\sigma }_{22}\right)}^{2}$$

In which ${\sigma }_{11}$ and ${\sigma }_{22}$ are the average stress in the longitudinal and vertical directions. This equation shows that the output electrical power of an auxetic structure is enhanced through the same sign of ${\sigma }_{11}$ and ${\sigma }_{22}$ against the plain structure, in which, ${\sigma }_{11}$ and ${\sigma }_{22}$ have different signs. (2) Power density is enhanced as a result of stress concentration closed to the corners and geometrical singularities. We have constitutive equations for the piezoelectric material as^44^

2 $${S}_{1}={s}_{11}^{E}{T}_{1}+{d}_{31}{E}_{3}$$

3 $${D}_{3}={d}_{31}{T}_{1}+{\varepsilon }_{33}^{T}{E}_{3}$$

in which ${S}_{1}$, ${T}_{1}$, ${E}_{3}$ and ${D}_{3}$ represents, the strain, the stress in the longitudinal direction of the piezoelectric element, the electric field and charge density respectively. ${s}_{11}^{E}$, ${d}_{31}$ and ${\varepsilon }_{33}^{T}$, stands for the electric compliance in a constant electric field, piezoelectric strain constant and the dielectric constant under constant stress, respectively. Considering the above constitutive equations, it is seen that, by increasing ${T}_{1}$ because of stress concentration, the charge density ${D}_{3}$ will be enlarged. These two advantages of auxetic structures lead to amplified harvested power compared to plain resonators.

### Finite element modeling

**Figure 5:**
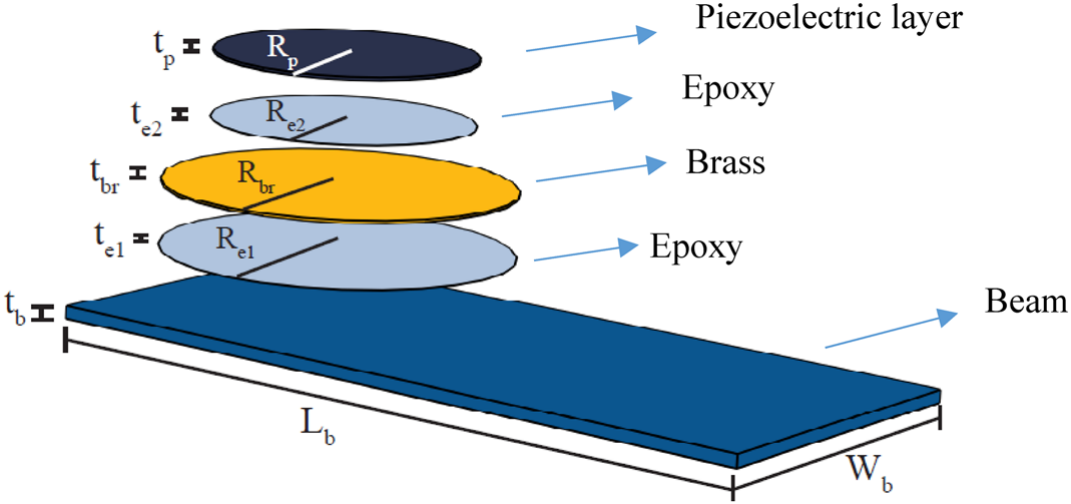
Different components of the FEM for PEH.

Finite element modeling (FEM) with COMSOL multiphysics is provided to examine the functioning of AEH-I, II and PEH in energy harvesting. Different components of the FE model are schematically shown in Fig. 5, consists of a PLA beam, piezoelectric element and epoxy layer for bonding (see Methods for more details). Table S1 and S2, summarize the geometrical and material properties of different elements.

### Numerical results

**Figure 6:**
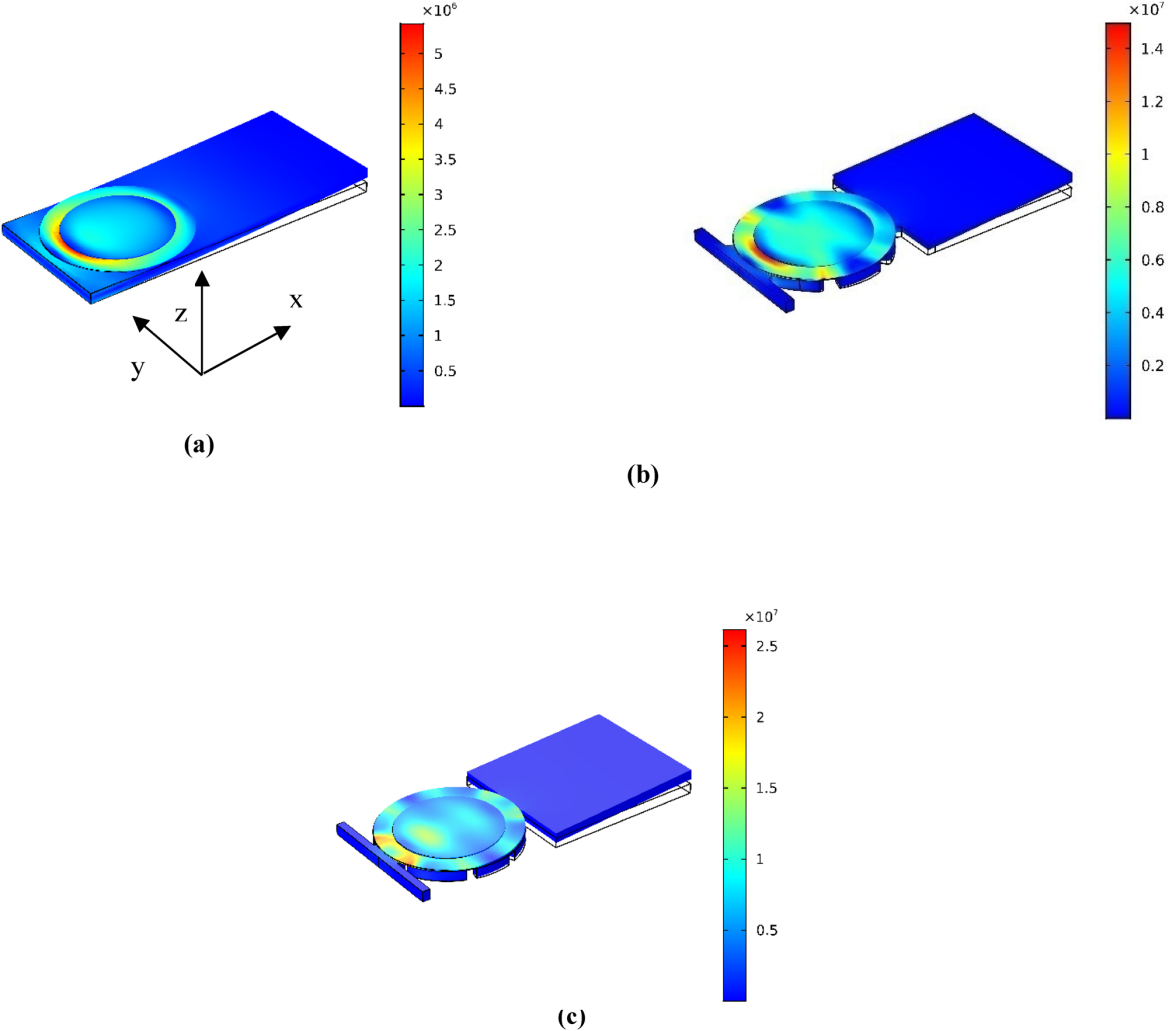
Total stress distribution in the energy harvesters **(a)** PEH, **(b)** AEH-I, **(c)** AEH-II.

Calculated total stress distributions $,$ in the PEH, AEH-I and AEH-II energy harvesters are illustrated in Fig. 6a–c, respectively. Frequency of the applied load is tuned on the main natural frequency of the beams which are $180$, $82$ and $59$ Hz for the PEH, AEH-I and AEH-II respectively. It is seen that the maximum stress is enhanced in the auxetic harvesters compared to the plain one. It is also more spread over larger areas. Table 1 summarizes the mean stress in the longitudinal (x) and vertical (y) directions for the PEH, AEH-I and AEH-II. The different sign of the average stress in these directions in PEH and same sign in the AEH-I and II is observed. As it was discussed, these two features are the main reasons why the auxetic substrates would enhance the output electrical power in piezoelectric buzzers.

**Table 1:** Mean stress values in longitudinal and transverse directions.

	${\stackrel{-}{{\varvec{\sigma}}}}_{11}$ (MPa)	${\stackrel{-}{{\varvec{\sigma}}}}_{22}$ (MPa)
PEH	− 0.39	0.11
AEH-I	− 0.99	− 0.64
AEH-II	− 1.47	− 0.22

**Figure 7:**
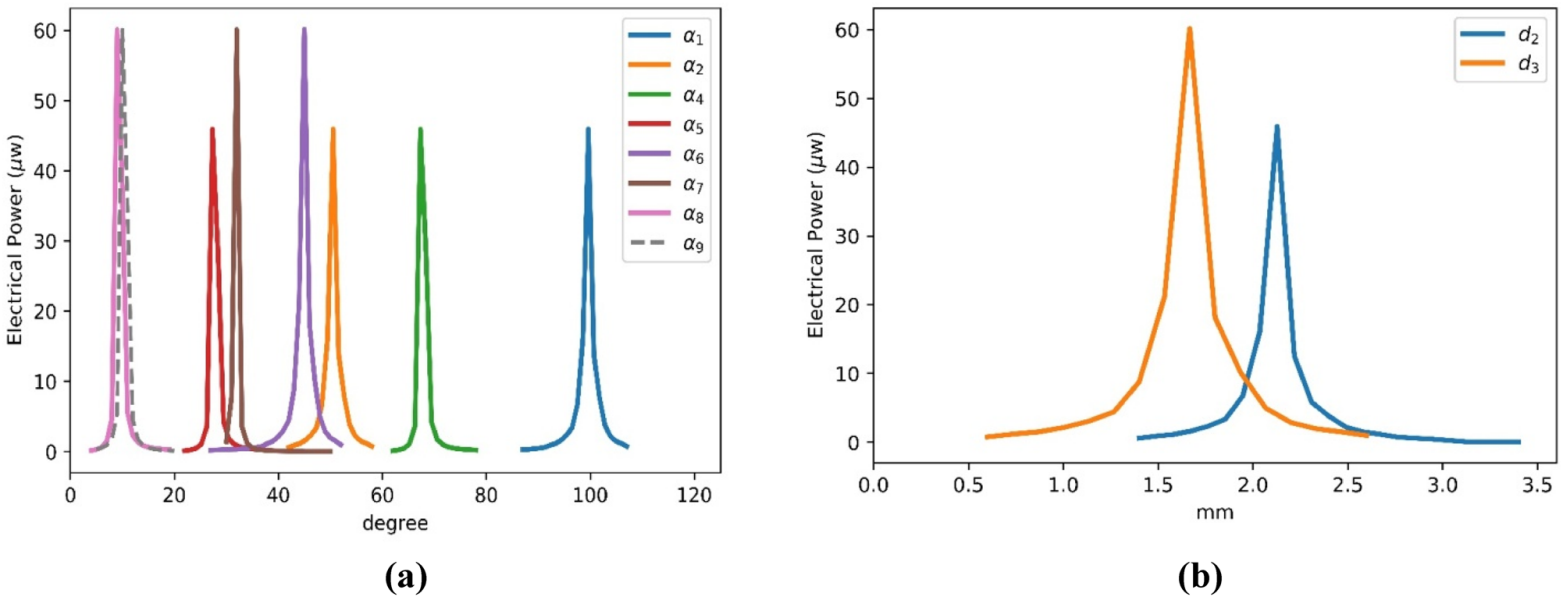
Effect of geometrical parameters on the harvested power of AEH-I and AEH-II, ${d}_{1}=1.5 \mathrm{mm}$**(a)** angles, **(b)** lengths.

Frequency tuning is the most important factor in any vibration energy harvester design. Accordingly, the design parameters should be optimized to tune the natural frequency of the harvester to the frequency of the source to harvest maximum electrical power. Effects of the geometrical parameters of the auxetic pattern on the harvested electrical power, which is calculated at optimal load condition, are illustrated in Fig. 7a,b. It is seen that the reported set of geometrical parameters for AEH-I and AEH-II in Table S1 are the optimum ones which lead to the maximum output electrical power at natural frequencies of AEH-I ($82$ Hz) and AEH-II ($59$ Hz). These set of optimal parameters are utilized for fabrication of the auxetic harvesters.

**Figure 8:**
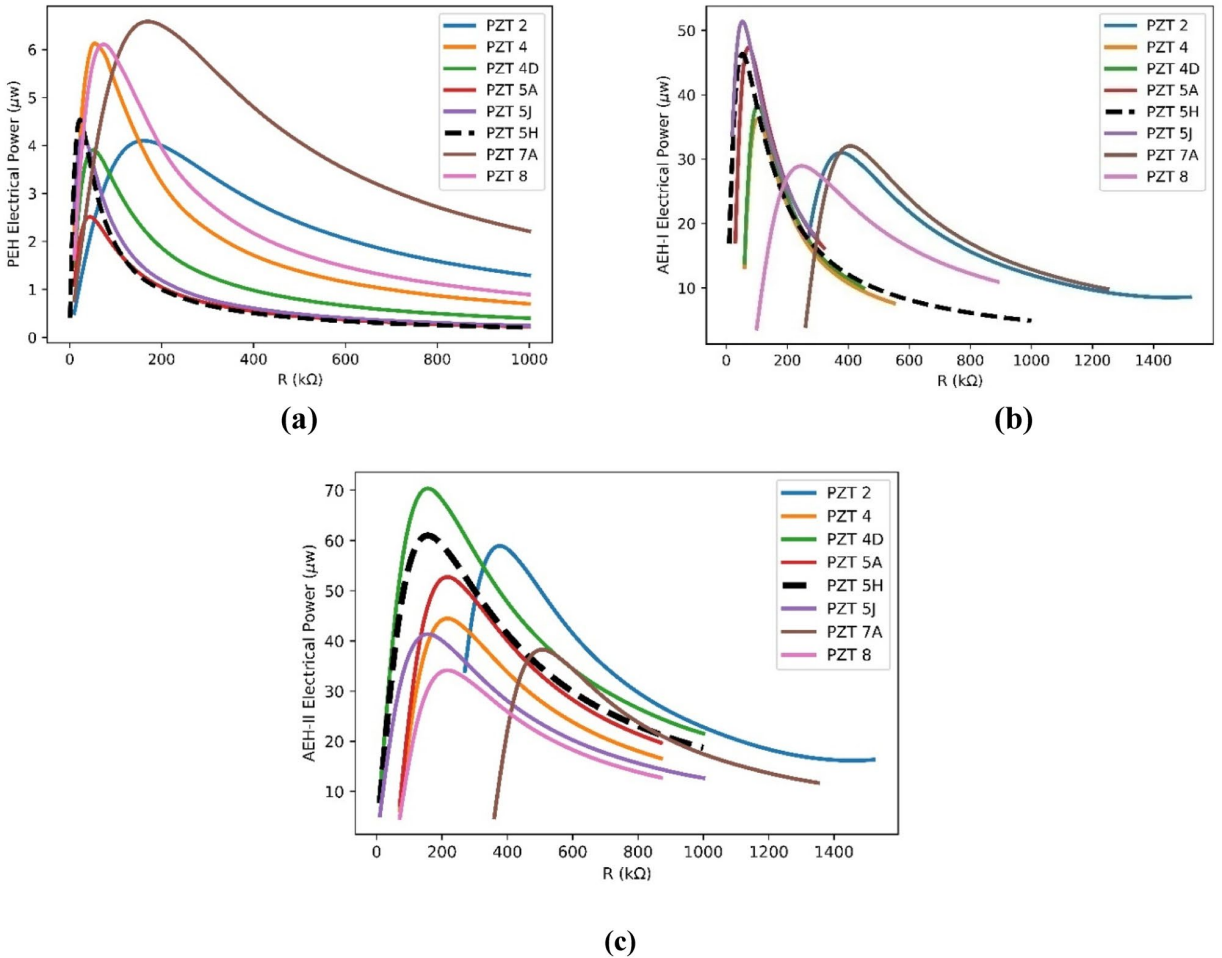
Effects of the PZT type on the generated power **(a)** PEH, **(b)** AEH-I, **(c)** AEH-II.

In order to investigate how the PZT type (PZT 2, 4, 4D, 5A, 5J, 5H, 7A, 8) would influence the generated power, different materials are explored in the FE model. Generated powers are presented in Fig. 8a–c. It is seen that PZT-7A, 5J and 4D, lead to the maximum harvested power in PEH, AEH-I and AEH-II, respectively. Results of the PZT 5H (the one used in experiment) is highlighted with black dashed line. Changing PZT material has a negligible effect on the resonance frequency as its size is small compared to the whole resonator. In case of having the plain geometry, harder piezoelectric materials normally lead to higher harvested power^44^. This is why PZT-7A, 8 and 4 with higher elastic constants, respectively, generate higher electrical power for the plane substrate in our calculation (Fig. 8a). However another key factor is the stress distribution applied to the PZT element. In other words the harvested power of a the piezoelectric element under vibration is related to piezoelectric properties and stress distribution^42^ that is $$Power\propto \frac{{{d}_{31}}^{2}}{{\varepsilon }_{33}}{\left({\tilde{\sigma }}_{11}+{\tilde{\sigma }}_{22}\right)}^{2}$$. This means that in addition to the material property ($${d}_{31})$$, combination of $${\left({\tilde{\sigma }}_{11}+{\tilde{\sigma }}_{22}\right)}^{2}$$ also plays important role in output generated power. Based on this fact, effect of the geometry of the auxetic design and its consequent stress distribution is mixed to hardness effect. Figure 8b,c are showing results of these combinational effects. In other words, in case of non-plain substrates, calculations should be carried out case by case and a general conclusion on the hardness effect is not still valid.

**Figure 9:**
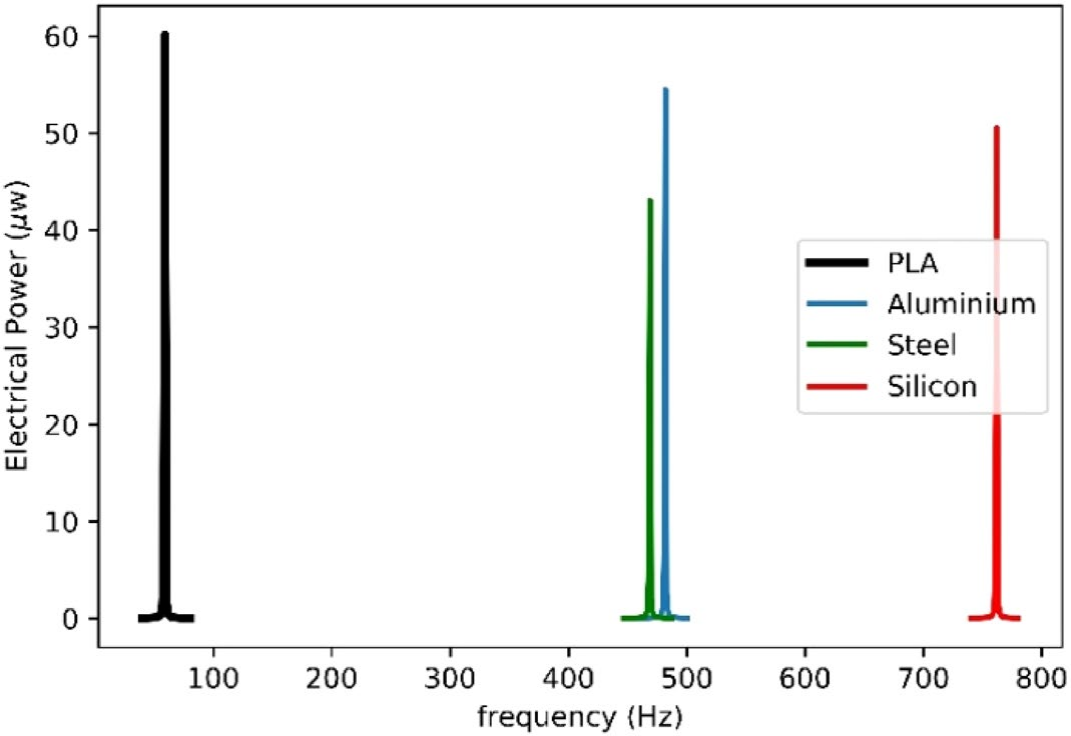
Effect of the substrate material on the AEH-II harvested electrical power.

Moreover, the effect of substrate’s materials is explored for AEH-II. Four different substrates, PLA, aluminum, steel and silicon are taken as the substrate and the corresponding harvested electrical powers with respect to the frequency are shown in Fig. 9. It is seen that the material with lower Young’s modules leads to higher harvested power with the same excitation and also, the natural frequency of the energy harvester would be altered by different substrate’s material.

### Experimental validation

**Figure 10:**
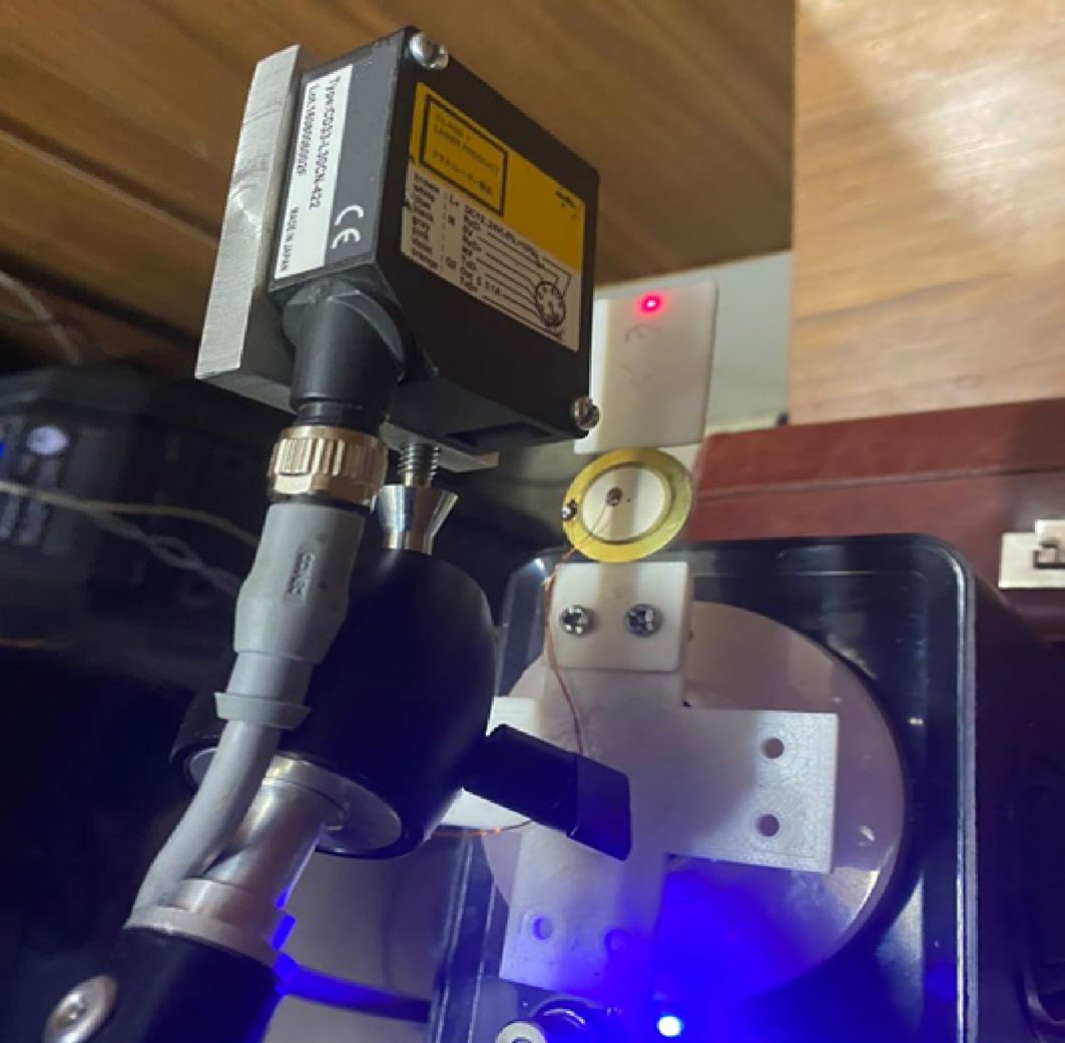
Top view of the experimental setup for the vibration energy harvesting system.

**Figure 11:**
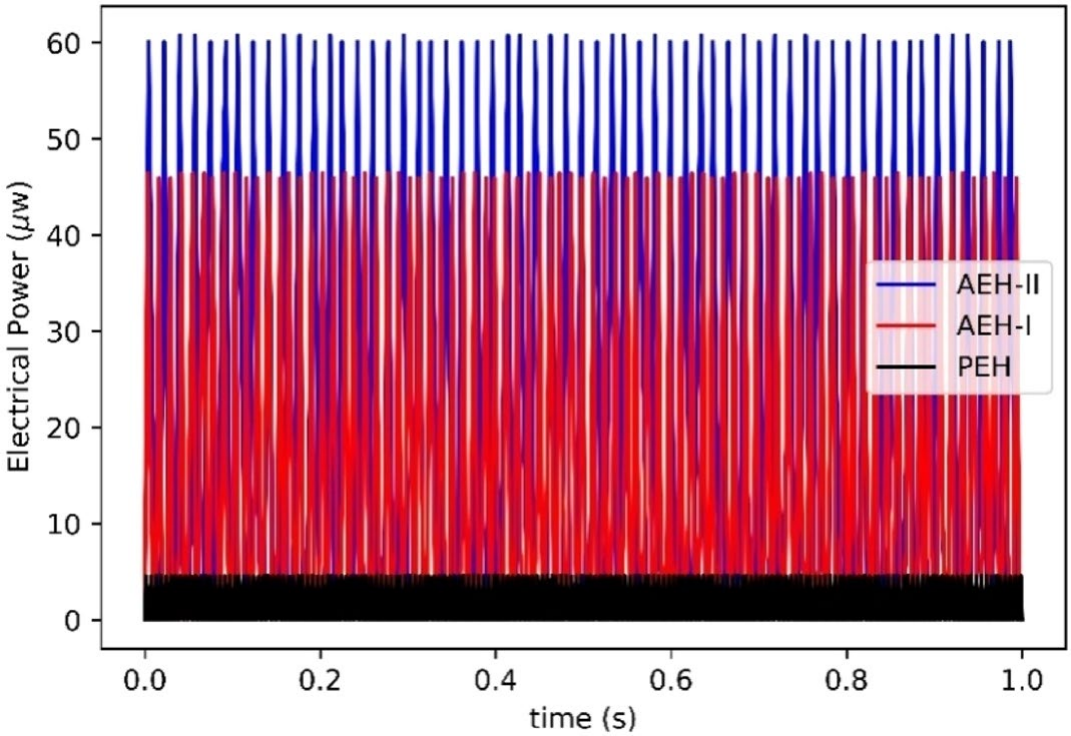
Experimental extracted power from auxetic and plain energy harvesters.

Figure 10 shows the experimental setup for the vibrational energy harvesting system (see methods for more details). Figure 11 shows time-history of the experimental harvested electrical power for the PEH, AEH-I and AEH-II. These results are for the optimum parameters in auxetic structures. It is seen that the AEH-II and AEH-I amplify the maximum and RMS value of the harvested electrical power by a magnification factor of $10.2$ and $13.3$ respectively.

**Figure 12:**
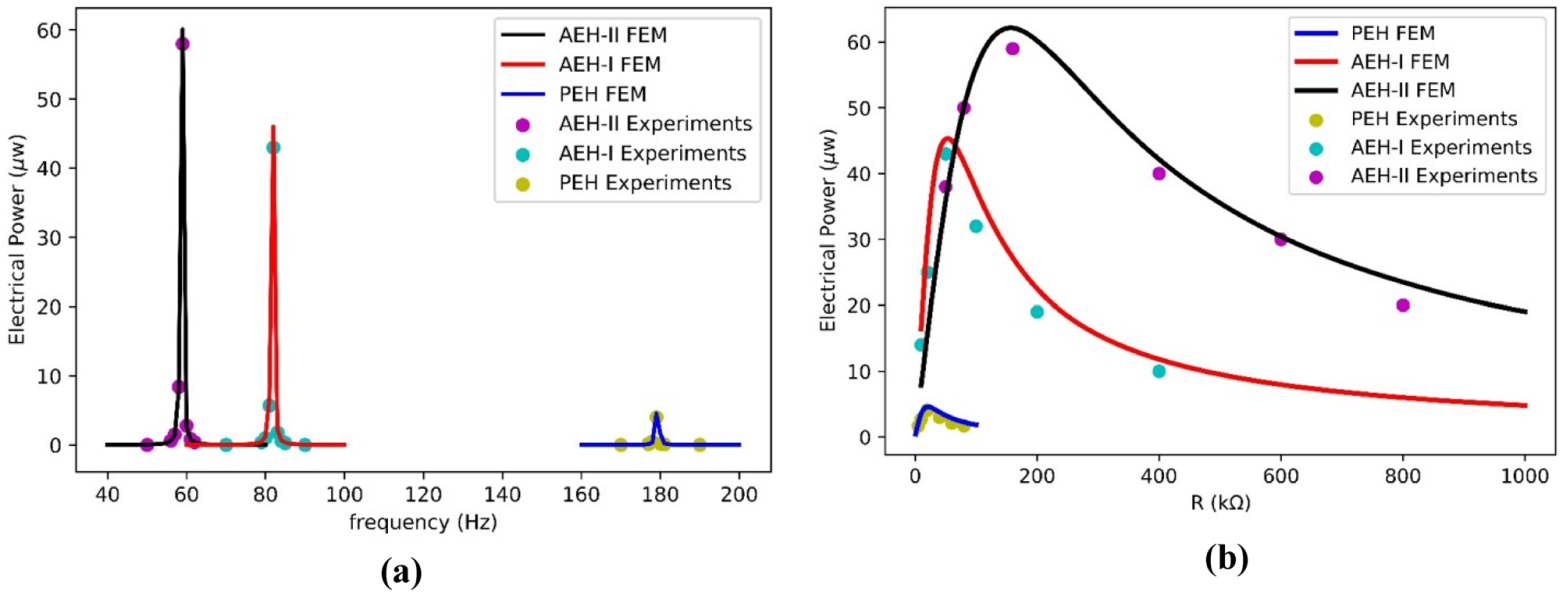
Numerical and experimental harvested power **(a)** frequency response, **(b)** variation against electrical resistance.

By comparing the harvested electrical power with respect to frequency value in model and experiment, the FE model is verified. Figure 12a illustrates the harvested power of PEH, AEH-I and II in model and experiments. The PEH, AEH-I and AEH-II are capable of harvesting $$4.5$$, $$45.9$$ and $$60.1$$$$\mathrm{\mu w}$$ electrical power, respectively. Furthermore, the effect of electrical resistance on the harvested electrical power from energy harvesters is shown in Fig. 12b. The matching impedance for the energy harvester is related to the operating frequency as well as piezoelectric parameters. Although the piezoelectric buzzers are the same for three energy harvesters in this study, however the optimum electrical resistance values for energy harvesters are different since the harvesters work in different working frequencies. The optimum electrical resistance for PEH, AEH-I and AEH-II is calculated to be $$20$$, $$50$$ and $$160$$$$\mathrm{k\Omega }$$, respectively. A good correlation between the FEM and experimental results is observed. Amplified electrical power is again seen in the model and experimental results. It is found, beside the power magnification, the auxetic designs works at frequencies very much lower (55% in AEH-I and 67% in AEH-II) than the plain conventional resonator. It is also found that the auxetic designs are more robust against variation of the optimum resistance.

Common environmental vibrational sources and human motions as desirable sources for generating electrical power are in low frequency range^45,46^. However, designing a low frequency energy harvester package with piezoelectric ceramics is challenging since these elements have high natural frequencies^47,48^. Structural vibrations for instance in bridges are also normally classified as low frequency vibrations. This means that self-powered sensors in condition monitoring of such structures are faced to low-frequency vibration for power extraction. Generally, designing a proper low-frequency vibration energy harvester is complicated from structural point of view^49^. Moreover, the generated power from vibrational energy harvester is proportional to the operating frequency which means that low frequency energy harvesters normally work at low power density^50^. These facts emphasize on the significance of power enhancement at low frequency range. Advantage of the proposed solution is that such power enhancement is achieved through auxetic structures without any need to material modification or extra mechanical or electrical elements.

## Discussion

Novel circular auxetic structures for enhancement of piezoelectric energy harvesting were proposed and tested in this paper. Finite element modeling was applied to investigate different important parameters and their effects on the performance of auxetic resonators in energy harvesting. Role of frequency, electrical resistance and geometrical parameters of the auxetic structures on the harvested electrical power were assessed. It was shown that there is an optimum magnitude of resistance and set of geometrical parameters of auxetic harvesters through which the maximum electrical power could be harvested. The proposed FE model is verified by mesh convergence criterion and experimental results. We could gain a magnification factor of $10.2$ and $13.3$ for AEH-I and AEH-II compared to the PEH in harvested electrical power. It was found that this superior performance is almost independent from the type of PZT material. Lower weight and resonance frequency of the system are two other side benefits. Outcomes guarantee potential, advantage and flexibility of the concept since it does not need any additional mechanical or electrical elements and can be easily applied to inexpensive PZT buzzers. This novel idea of auxetic structures for energy harvesting can be utilized in different mechanisms of piezoelectric energy harvesting for different applications particularly in self-powered sensor networks.

## Methods

### Modeling

COMSOL multiphysics 5.5 is utilized in order to model the energy harvesters. “Solid Mechanics Physic” is applied to model the mechanical properties of the beams, epoxy and piezoelectric elements. The stress tensor is then fed into the charge transformation module in piezoelectric element. Fix boundary condition at one end makes a cantilever beam and a body load of 0.003 $N$ is applied. Thin elastic layer boundary condition is used to model the epoxy properly^42^. The “solid mechanics physic” is coupled to “electrostatic physic” in which the charge conservation domain is set to be the piezoelectric element. In order to calculate the generated electrical power by the piezoelectric element, the generated voltage is applied to the two pins of an electrical resistance in which the dissipated power is considered as the harvestable electrical power.

In the finite element model, two different simulations are carried out. Within the modal analysis we calculate the natural frequency of the plain and auxetic energy harvesters to specify the frequency in which the maximum electrical power is harvestable. Subsequently, a frequency domain analysis is carried out to excite the energy harvesters at different tones and calculate the generated electrical power.

**Figure 13:**
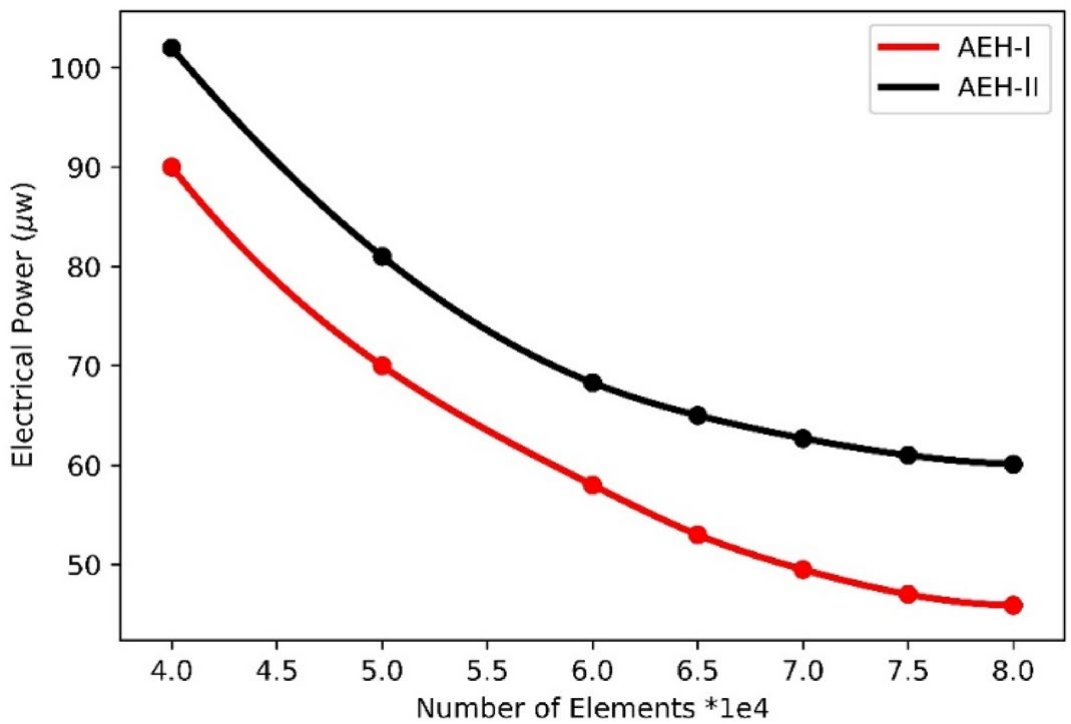
Numerical convergence of results with respect to the included mesh elements number.

The finite element model is verified by experimental results (Fig. 12) and mesh convergence criteria (Fig. 13). It is seen that for a sample case study, increasing the number of included meshes, the electrical harvested power of AEH-I and AEH-II are monotonically converged to the 45.9 and 60.1 μw, respectively.

### Fabrication of beams

These energy harvester substrates are made of polylactic acid (PLA) and fabricated by 3D printer (Trust-L-Pro model) with accuracy of 20 microns. In order to fabricate the substrates, the design CAD files are constructed using COMSOL designing tools. Optimal geometrical parameters are employed in fabrication of the resonators.

### Measurement

Fabricated resonators with attached piezoelectric buzzers are mounted on the shaker and excited in different frequencies. The ground and terminal of each piezoelectric element are connected to a rheostat to measure the dissipated power. In order to extract the signal from the piezoelectric elements and preparing it for further processing, we have used and Arduino Mega 2560 board based on ATmega 2560 microcontroller. One wire must connect the rheostat to the ground port of the Arduino, and the other should be connected to one of the analog ports. Using the *analogRead* property of the Arduino, the input analog voltage will be converted to digital signal by analog to digital converter of the Arduino. This results in an integer value between 0 to 1023 from the input analog voltage which is between 0 and the operating voltage of the Arduino board (5 V for Arduino Mega 2560). Utilizing LabView interface for Arduino toolkit, we are capable of building graphical interfaces to implement a real-time data acquisition and analysis system based on the Arduino signal. This system enables a precise control on the input signal and its characteristics. Vertical displacements of the cantilever resonators are measured by a laser vibrometer with accuracy of two microns.

## Supplementary information

Supplementary Information.

Supplementary Video 1.

Supplementary Video 2.
