# Supplementary material for: Study in circular auxetic structures for efficiency enhancement in piezoelectric vibration energy harvesting
Source: Sci Rep. 2020 Oct 1;10:16338. doi: 10.1038/s41598-020-73425-1 (PMC7531003; doi:10.1038/s41598-020-73425-1)

**Supporting Information**

Study in Circular Auxetic Structures for Efficiency Enhancement in Piezoelectric Vibration Energy Harvesting

Pejman Eghbali^1^, Davood Younesian^1^, Armin Moayedizadeh^1^, Mostafa Ranjbar^2^

^1^School of Engineering, Iran University of Science and Technology, Tehran 16846-13114, Iran

^2^Department of Mechanical Engineering, Ankara Yildirim Beyazit University, Ankara, Turkey

**Table S1:** Geometrical properties used in the model

| Part | Parameter | Symbol | Value | Unit |
| --- | --- | --- | --- | --- |
| Cantilever Resonator | Length | $L_{b}$ | 60 | mm |
|  | Width | $W_{b}$ | 27 | mm |
|  | Thickness | $t_{b}$ | 2 | mm |
| Epoxy Layer 1 | Radius | $R_{e1}$ | 13.5 | mm |
|  | Thickness | $t_{e1}$ | 30e-3 | mm |
|  | Bonding strength | $k$ | 200 | GN/$m^{3}$ |
| Brass | Radius | $R_{br}$ | 13.5 | mm |
|  | Thickness | $t_{b}$ | 0.22 | mm |
| Epoxy Layer 2 | Radius | $R_{e2}$ | 13.5 | mm |
|  | Thickness | $t_{e2}$ | 30e-3 | mm |
|  | Bonding strength | $k$ | 200 | GN/$m^{3}$ |
| Piezoelectric layer | Radius | $R_{p}$ | 13.5 | mm |
|  | Thickness | $t_{p}$ | 0.2 | mm |
| Auxetic Region |  |  |  |  |
| AEH-I |  | $d_{1}$ | 1.5 | mm |
|  |  | $d_{2}$ | 2.1 | mm |
|  |  | $\alpha_{1}$ | 100 | deg |
|  |  | $\alpha_{2}$ | 50 | deg |
|  |  | $\alpha_{3}$ | 100 | deg |
|  |  | $\alpha_{4}$ | 69 | deg |
|  |  | $\alpha_{5}$ | 27 | deg |
| AEH-II |  | $d_{3}$ | 1.6 | mm |
|  |  | $\alpha_{6}$ | 44 | deg |
|  |  | $\alpha_{7}$ | 32 | deg |
|  |  | $\alpha_{8}$ | 9 | deg |
|  |  | $\alpha_{9}$ | 10 | deg |

**Table S2:** Material properties used in the model

| Material | Property |  | Value | Unit |
| --- | --- | --- | --- | --- |
| PLA | Density | $\rho_{PLA}$ | 1.23e3 | Kg/$m^{3}$ |
|  | Poisson’s Ratio | $\nu_{PLA}$ | 0.36 |  |
|  | Young’s Modulus | $E_{PLA}$ | 3 | GPa |
| Epoxy | Density | $\rho_{e}$ | 1250 | Kg/$m^{3}$ |
|  | Poisson’s Ratio | $\nu_{e}$ | 0.35 |  |
|  | Young’s Modulus | $E_{e}$ | 1 | GPa |
| Brass | Density | $\rho_{b}$ | 8490 | Kg/$m^{3}$ |
|  | Poisson’s Ratio | $\nu_{b}$ | 0.31 |  |
|  | Young’s Modulus | $E_{b}$ | 97 | GPa |
| Lead Zirconate Titanate (PZT-5H):  (Piezo-Element) | Density | $\rho_{p}$ | 7500 | Kg/$m^{3}$ |
|  | Compliance Matrix | $s_{11}^{E}$ | 1.65e-11 | 1/Pa |
|  |  | $s_{12}^{E}$ | -4.7e-12 | 1/Pa |
|  |  | $s_{22}^{E}$ | 1.65e-11 | 1/Pa |
|  |  | $s_{13}^{E}$ | -8.45e-12 | 1/Pa |
|  |  | $s_{23}^{E}$ | -8.45e-12 | 1/Pa |
|  |  | $s_{33}^{E}$ | 2.07e-11 | 1/Pa |
|  |  | $s_{44}^{E}$ | 4.35e-11 | 1/Pa |
|  |  | $s_{55}^{E}$ | 4.35e-11 | 1/Pa |
|  |  | $s_{66}^{E}$ | 4.26e-11 | 1/Pa |
|  | Coupling Matrix | $d_{31}$ | -2.74e-10 | C/N |
|  |  | $d_{32}$ | -2.74e-10 | C/N |
|  |  | $d_{33}$ | 5.93e-10 | C/N |
|  |  | $d_{24}$ | 7.41e-10 | C/N |
|  |  | $d_{15}$ | 7.41e-10 | C/N |
|  | Relative Permittivity | $\varepsilon_{11}$ | 3130 |  |
|  |  | $\varepsilon_{22}$ | 3130 |  |
|  |  | $\varepsilon_{33}$ | 3400 |  |

**Supporting Movie S1**

Video showing the conventional cantilever-type vibration energy harvester with plain substrate,

the system is directly powering LEDs for sinusoidal input with frequency of 180 Hz equal to the resonance frequency of the plain system.


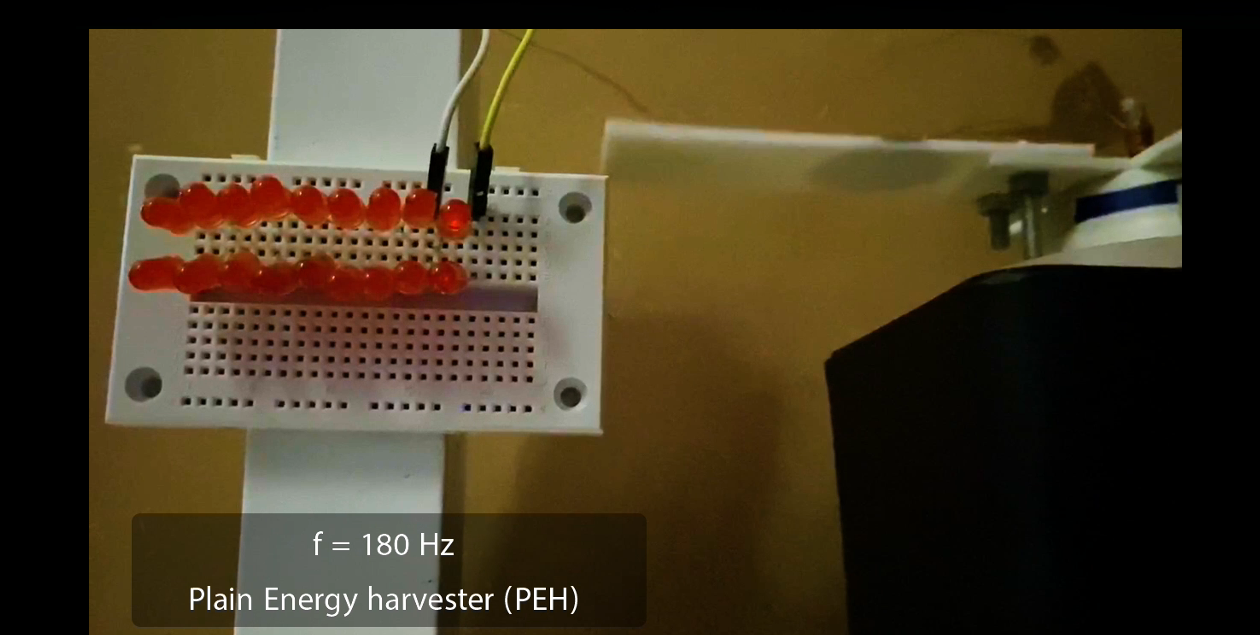


**Supporting Movie S2**

Video showing the conventional cantilever-type vibration energy harvester with auxetic substrate,

the system is directly powering LEDs for sinusoidal input with frequency of 59 Hz equal to the resonance frequency of the auxetic system with geometry of AEH-II.


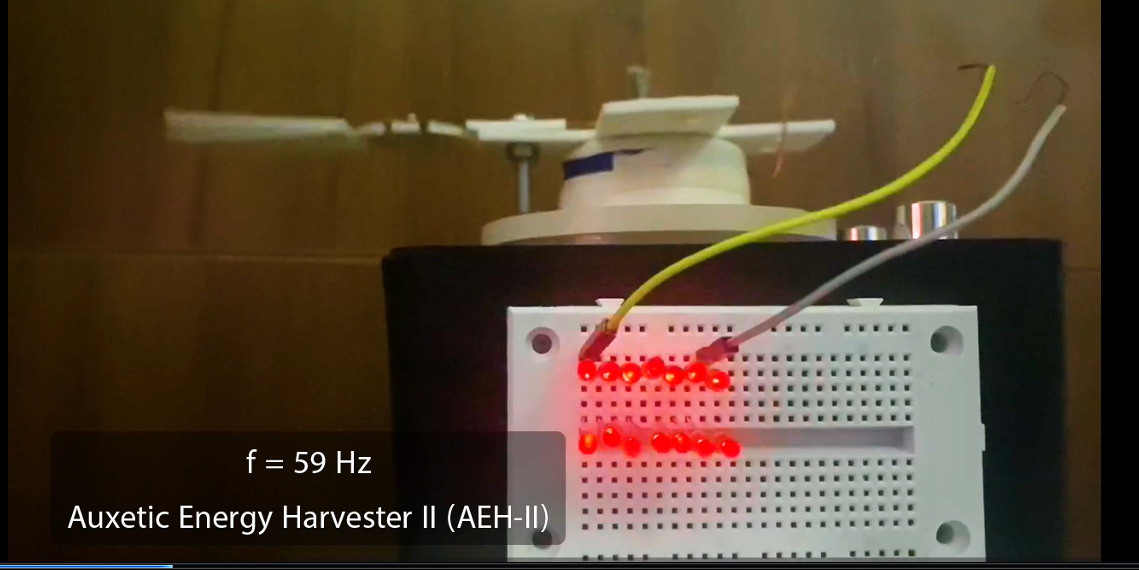

Supplement: Supplementary file 1 — Supplementary Information. [file 41598_2020_73425_MOESM1_ESM.docx]
